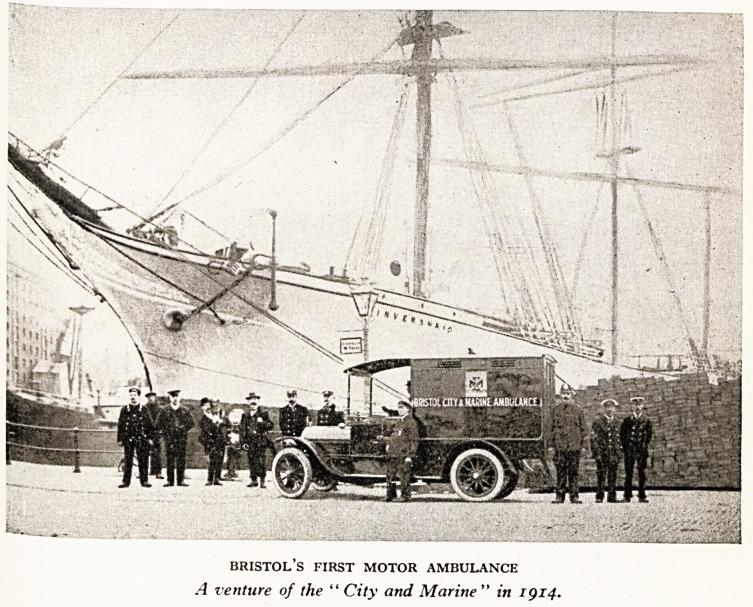# Some Surgical Milestones

**Published:** 1951-01

**Authors:** W. A. Jackman

**Affiliations:** Surgeon, United Bristol Hospitals


### The Bristol
### Medico-Chirurgical Journal

*A Journal of the Medical Sciences for the*

### West of England and South Wales

*"Scire est nescire, nisi id me*
*Scire alius sciret."*

JANUARY, 1951

### SOME SURGICAL MILESTONES

The Presidential Address, delivered on Wednesday, 11th October, 1950, at the opening of the Seventy-second Session of the Bristol Medico-Chirurgical Society

BY

W. A. JACKMAN, *T.D.*, F.R.C.S.

*Surgeon, United Bristol Hospitals*

In this address I propose to remind you of some important milestones on the highway of surgery, now accepted as a matter of course, but which indeed have changed the whole trend of surgical practice. In order to give our younger members some idea of the period which I propose to cover perhaps I ought to say that I first visited the Bristol Royal Infirmary some time in the year 1911. As a second year student burning to get a taste of what was to come, I ventured into the Grieg Smith theatre where Dr. James Swain was amputating a breast. The anaesthetic, chloroform and ether, was administered by the late Dr. A. L. Flemming; and some 12 feet distant from the anaesthetist's table the flames of an open fire danced merrily in the grate, occasionally stoked by the theatre sister, Sister Fanny Gross. Subsequently on that day, I remember seeing Dr. Swain enter his carriage, a Victoria, a type very popular with the medical profession in those days.

With this vivid impression still in my mind, you may understand why I feel it imperative to refer briefly to the change in the methods of transport over these years. For what we now accept as a foregone conclusion, rapid, faultless transmission from place to place, has but recently evolved from the horse age, through a period of distinct mechanical uncertainty to that of the present day.

Not only has this revolution had a profound effect on the doctor's perambulation but also on that of the patient. My contemporaries will remember the snappy horse ambulance owned and run by the City and Marine, which rendered yeoman service to the sick and injured in and around this city; a service in which St. John's also played their valuable part. From this type of vehicle there evolved the motor ambulance. The first of its kind in Bristol was also a venture of the City and Marine Ambulance Corps in the year 1914. (Plate 1.) From this we have passed to the ambulance of the present day, which not only shows a marked advance in design and efficiency but seems to have exceeded in fecundity that of the inhabitants of the district. For it is now unusual to drive more than a few hundred yards without passing a vehicle labelled with its sinister legend: whereas, but a year or two ago, the occasional appearance of an ambulance made one wonder what had happened and where.

In the early days of which I speak it was quite usual for urgent operations to be carried out in the patient's home because of the difficulty of moving the " very ill " quickly and without undue disturbance, and because the patients themselves, their relations or both, refused admission to what they described as " those dreadful places ".

I would like also to remind you of the profound changes in methods of communication, for the telephone has now replaced the footslogging messenger and its increasing use must have resulted in a more prompt handling of the surgical emergency. In Bristol in 1912 there were 7,000 lines: to-day over 29,000.

### 1910–20

Commencing in this decade I wish now to refer to some revolutionary surgical procedures.

PLATE I

BRISTOL'S FIRST MOTOR AMBULANCE

*A venture of the "City and Marine" in 1914.*

*Blood Transfusion* was not new. It had most certainly been attempted in Great Britain from time to time since 1604, at first using the blood of some animal. Sir Geoffrey Keynes in his book on the subject describes the first transfusion of human blood in this country by Blundel and Cline on 22nd December 1818. There seemed little hope of even reasonable success until 1901, when Lansteiner in Vienna and Shattock in London recognised in the blood the presence of agglutinins and iso-agglutinins which lead to the subsequent division of the blood of individuals into four groups. It took some time for all this to filter through and become stabilised; and it would be very nearly correct to say that until the principles involved were universally recognised and adopted in practice, almost as many lives were lost through blood transfusion as were saved by it.

It was not until we were half-way through the 1914–18 war that blood transfusion really got going, some fifteen years after the recognition of the blood groups. The methods employed for giving it were crude and often complicated and remained so until the perfection of the drip method which we use to-day. Our path is made still easier by the establishment of the blood bank and in this area we must thank Sir Lionel Whitby for his initial organisation.

I shall never forget the difficulty of obtaining donors in those early days; in the services it was not so bad, but in civil practice often very difficult. Housemen and medical students were ready volunteers, for it was not long before the hospitals were willing to pay for blood at the rate of three guineas per pint: the " group 4's " were thereby often saved the pawning of the microscope, and were willing to be bled white in the cause of financial stability. Later on the comparatively well patients, herniae and the like, were asked to volunteer and it was part of the duty of the S.R.O. to keep a list of the " universal donors " in the building. Still later we had a rota of citizen volunteers together with their addresses and telephone numbers. Thus was blood transfusion ushered in as a routine measure in medicine and surgery, a milestone that marked the way to the saving of countless valuable lives.

*The drip method* I have mentioned. We can hardly imagine hospital practice bereft of this simple procedure—drips into the veins, under the skin, into the rectum, the oesophagus and the stomach, and as a means of maintaining continuous suction.

*Analine dyes.* I have not the time to recall any other notable advance in surgical practice in the 1910–20 decade except perhaps the adoption of the analine dyes in the role of antiseptics. I remember two eminent Bristol surgeons, the late Mr. Ferrier Walters and Professor Rendle Short, as members of research teams for the study of the relative merits of mercurochrome and acriflavine. On the one hand Mr. Walters and his team, after extensive investigation, decided that mercurochrome was a most efficient antiseptic and that acriflavine was useless. On the other, Professor Rendle Short and his followers came to the conclusion that acriflavine was a valuable antiseptic whereas mercurochrome was best left in its more appropriate role as red ink. The reports were supported by adequate statistics. Needless to say, when I was H.S. to Mr. Ferrier Walters I used mercurochrome, and when filling a similar post for Mr. Short I used acriflavine. My impression was that the majority of our patients dealt very adequately with their infections.

### 1920–30

Those of us who can look back to the 1920's must agree that it was an era of " stunts and gadgets ". I do not feel that I must apologise for using these terms, for they are both in the English dictionary: " Stunt; Tour-de-force, special effort, display of concentrated energy, a turn of work, a course of action, an act, a performance, a trick. Gadget ; A small fitting or contrivance in machinery etc., a dodge or device." My own definition of a gadget would be " something with which you perform a stunt". To mention a few:

*Plaster of Paris* came into its own: and among its most valuable uses was that of immobilising limbs, the site of severe infections, osteomyelitis, open fractures and the like. Headed by an American surgeon, Winnett Orr by name, complete rest, semi-permanent bland dressings and maggots intrinsic and extrinsic became commonly used in practice and certainly marked one initial phase of vast improvement in the treatment of such cases.

*Skeletal Traction* was very lucidly described by the late

Professor Hey Groves in the British Journal of Surgery for 1928/9, together with his special device for the purpose.

*Diathermy and the Sucker.* This decade saw the birth of surgical diathermy and the sucker as articles of theatre equipment, now an essential part of operative technique in most fields. The first diathermy apparatus for operative surgery was made by the Genito-Urinary Manufacturing Company in 1924, at the request of the late Frank Kidd. The apparatus is not without its dangers. We soon learnt that it must not be used in close proximity to bone, cartilage and certain of the vital organs, and should be treated with great respect in or near the hollow viscera and at the skin edge.

My research into the origin of the sucker is not very precise. It seems gradually to have evolved from that produced in the world of hydrodynamics to the present-day motor suction pump, which is almost too powerful. In fact, this violent suction can be lessened by the insertion in the circuit of a glass 'Y' piece; and this will certainly prevent soft tissue being sucked violently into the nozzle, and so avoid possible injury to vulnerable structures and friable tissues. The danger of using the sucker as an alternative to the swab is readily apparent, for serious haemorrhage may be overlooked. Where this catastrophe is a possibility some scheme must be adopted whereby careful watch is kept on the amount of blood lost.

*Ryle's Tube.* In 1926 the late Dr. John Ryle, who died only last March, published a book called "The Gastric Function in Health and Disease". In this he described gastric analysis, the fractional test meal, and his own modification of the Rehfus tube—Ryle's tube. Since that day this simple device has not only served its original and designed purpose. It must have saved thousands of lives; or ameliorated the lot of those inevitably condemned to death during their last days or hours. It has proved of value in pre-operative preparation and post-operative care, in the treatment of ulcers and other gastric conditions. There can be few surgeons who have not cause to be grateful to this distinguished physician for giving us so valuable an addition to our armamentarium.

The 1920's were also marked by the development and elaboration of illuminated endoscopes for peering into the body cavities through natural and other orifices, and indeed for the performance of surgical manoeuvres thereby.

Truly an era of Stunts and Gadgets.

The President next dealt with the years 1930–40 which he considered remarkable chiefly for the introduction of chemotherapy and antibiotics. He described the discovery, development, uses and abuses of the various agents.

### 1940–50

We can regard the 1940's as the age of "buttoning-up", the crossing of 't 's, the dotting of 'i 's and the rounding off of jagged corners. Infusions, transfusions, drips, suction, antibiotics, instruments and appliances have all been and are being improved and developed by a gradual process of evolution. We see advances step by step in every phase and aspect of surgical practice, so that today surgery bears little resemblance to that which was practised 40 years ago, except for the use of the knife and the haemostat.

Vast developments are taking place in the allied sciences. Radioactive isotypes, tracer substances and the like are evolving for our use in diagnosis and treatment. Operations are being undertaken which, but a few years back, were deemed impossible, and the interdependence of the various sciences suggest an era of combined operations.

I must refer to the tremendous forward strides made in the realms of anaesthesia. Earlier I spoke of an anaesthetist at the B.R.I., Dr. A. L. Flemming, now unhappily gone from our midst. I shall always remember him as the anaesthetist who gave as a definition of a surgeon "One who takes life easily". The apparatus which he used was simple in the extreme, a mask and two bottles. Yet he and his colleagues of those days gave most excellent anaesthetics and I often wonder if the anaesthetic risks were not rather in the minds of surgeons, and that hasty and rough handling contributed largely to those complications which did arise. Now the anaesthetic apparatus has become more complicated, and the impressive nature of the modern "set-up" compels our awed respect, for who could doubt that from this emanates the very essence of controlled and perfect narcosis.

I feel I must refer to the almost incredible improvement in the surgery of infancy and early childhood, throughout these years. With appropriate pre-operative preparation and post-operative management a nursling will survive quite major surgical procedures. Conrad Rammstedt of Münster should be remembered as a pioneer in infant surgery. He discovered by accident a simple operation for the cure of an otherwise fatal disease, and thereby both directly and indirectly preserved the lives of many hundreds or thousands of infants who have thrived and matured to become valuable citizens.

In my student days we used to see infants suffering from a disease called Marasmus; Galen, Marasmos, " wasting away ". They did. There can be little doubt that many such children suffered from unrecognised pyloric stenosis. A cure having been devised, it became imperative to recognise the condition and submit the patients for treatment. In Bristol in 1914 126 infants were notified as having died of marasmus, a death rate of 16·3 per 1,000 live births. In 1944 there were 6 such deaths (0·8 per 1,000) and in 1949 there were no deaths registered from this disease.

I now wish to refer to the late Thomas Carwardine who retired from the staff of the B.R.I. in 1926, and died about three years ago. He was a surgeon renowned for his technical skill; those of us who remember his work will recall each of his operations as a poem of perfect precision. He gave to surgery the anastomosis forceps. His activities covered the whole field of surgery and he was capable of performing any of the operations of surgery with equal facility. I cite him merely as a representative of any surgeon of his day in the top rank of his profession. He practised in the days when this country was probably at its wealthiest, when there was no shortage of man-power, money or material, when a fee of one guinea was paid as a golden sovereign and a silver shilling. Now, when we are told we have never been poorer in every possible respect, when manpower is short and a guinea represented by a worthless piece of paper and a disc of cupro-nickel, the field which he covered requires:

The Gynaecologist,
The E.N.T. Surgeon,
The Orthopaedic Surgeon,
The Plastic Surgeon,

The Neuro-Surgeon,
The Genito-Urinary Surgeon, and
The General Surgeon or Herniotomist and Appendicectomist.

There are signs that still more "high specialisation" is imminent. We have never been poorer, yet we live more and more extravagantly.

We see then, over these four decades vast improvements and advances in the field of surgery. Yet we must universally regret and admit that our ignorance of the aetiology, course and treatment of cancer remains little short of appalling, a state of affairs which is certainly not for want of effort. The best brains of our profession are daily directed towards the solution of the problem in all its aspects, so far without result. To refer to our own city, in Bristol the death rate from cancer has steadily risen from 113 in 1914 to 187 per 100,000 of the population in 1949: when 823 persons were registered as having died of cancer in a civilian population of 439,740. Perhaps the 1950's will bring us greater hope.

## Figures and Tables

**Figure f1:**